# Online misinformation can distort witnesses’ memories. Analysis of co-witness discussions using an online version of the MORI-v technique

**DOI:** 10.3389/fpsyg.2023.1239139

**Published:** 2024-01-17

**Authors:** Magdalena Kękuś, Malwina Szpitalak, Romuald Polczyk, Krystian Barzykowski

**Affiliations:** ^1^Faculty of Psychology in Kraków, SWPS University, Kraków, Poland; ^2^Institute of Psychology, Jagiellonian University, Kraków, Poland

**Keywords:** memory conformity effect, memory distortions, co-witness discussion, eyewitness testimonies, MORI technique

## Abstract

**Introduction:**

The memory conformity effect occurs when people witness a given incident and then talk to each other about it, and the statement of one person affects the memory account of another person with respect to that incident. The main objectives of this experiment were (1) to examine the effectiveness of a modified version of the MORI-v technique in inducing the memory conformity effect and (2) to investigate how the manner in which participants discuss the observed event influences the magnitude of this effect. In general, the modified online MORI-v technique consists of the following main elements: (1) *original material*, that is, two versions of a short film which are identical except for certain critical details; for example, in one version, a thief puts on a red cap, but in the other version it is black; (2) the *collaborative recognition test*, that is, a discussion about the original material which leads to mutual misinformation; and (3) an *individual recognition test* that checks the effect of the discussion on the memory account of the original material.

**Methods:**

A total of 72 participants (36 pairs) aged 18–54 took part in the research. Participants were tested using the online MORI-v technique: They were familiarized with the original material on their computers at home, and then they talked about it via a video communication app and completed an individual recognition test on their computers. Importantly, the discussions were recorded and analyzed in detail after the experimental session.

**Results and discussion:**

Using the online MORI-v technique, the effect of memory conformity was demonstrated, that is, in the individual recognition test, the proportion of correct answers to questions about discussed details (related to misinformation) was lower than the proportion of correct answers to questions about non-discussed details. It was also demonstrated that if one participant introduced misinformation during the discussion about a particular item and the other did not question it, the latter’s answer to that item during the individual recognition test was most often incorrect. However, if one participant introduced misinformation during the discussion about an item and the other questioned it, the latter’s answer about that item during the individual recognition test was most often correct.

## 1 Introduction

The memory conformity effect occurs when people witness a given incident (e.g., a crime) and then talk to each other about it, and the statement of one person affects the memory account of the other person with respect to that incident (Wright et al., 2000). For instance, a witness, despite seeing a criminal wearing a gray coat, testifies that the coat was brown as this was the information heard from a co-witness. Thus, this phenomenon may contribute to unreliable testimonies, which remain the leading cause of incorrect court decisions (Smith and Cutler, 2013). It should be stressed that such a mistake may have serious consequences, including the conviction of an innocent person or the acquittal of a guilty one (Greene and Loftus, 1984). Consequently, the memory conformity effect is the subject of a lot of research.

Various methods have been used to present participants with misinformation stemming from other persons. In a seminal experiment, Schneider and Watkins (1996) presented a list of words to pairs of participants (Experiment 1) or to pairs in which one member was a researcher’s accomplice (Experiment 2). The members of the pairs took turns to respond first. The second responses were strongly biased by the first responses. This effect has been replicated many times (e.g., Wright et al., 2000, 2005; Skagerberg and Wright, 2008, 2009; Thorley and Dewhurst, 2009, Experiment 3).

The above-described procedure consisted of two stages: Presentation of some original material and participants taking turns to respond to questions. An important extension of this paradigm that consists of three stages was proposed by Roediger et al. (2001; they called this paradigm “social contagion”). They studied pairs of people, one of whom was actually a confederate. The pairs watched household scenes, and during the following collaborative recall, the confederate introduced false answers in some cases. This resulted in “recalling” many of the suggested answers in a subsequent individual memory test. The fact that memory reports may be distorted as a result of wrong answers provided previously by a confederate has been replicated many times (e.g., Meade and Roediger, 2002; Wright et al., 2005; Allan and Gabbert, 2008; Zajac and Henderson, 2009; Thorley, 2013; Williamson et al., 2013; Szpitalak et al., 2015; Doughty et al., 2017; Calado et al., 2018, Experiments 1 and 2).

Another design used in memory conformity research consists of having two actual participants listening to or watching slightly different versions of the original material without knowledge of the differences. For example, in research by Gabbert et al. (2007), two participants were seated at computer desks with their backs to one another while they looked at pictures, which actually differed in some details. After a filler task, the participants then “recalled jointly” the pictures; in this way, they often misinformed each other because they were recalling details that were correct for only one of them. In a final performed individual memory test, fewer correct answers were given for critically discussed details. Other methods used to make the pairs of participants believe that they were watching the same video included, for example, having the participants watch the video one at a time “as there was only one monitor” (Bartlett et al., 2021) or separating them with a screen (Gabbert et al., 2003). Similarly, listening to different versions of an audiotape (Oeberst and Seidemann, 2014) and collaboratively recalling it resulted in biased answers in a subsequent individual memory test. In a rare example of an experiment outside the laboratory, Carlucci et al. (2011) had a confederate approach to small groups on a beach and interacted with one group member. Afterward, the experimenter asked the group members to identify the confederate in a target-absent line-up. Group members are more likely to conform to the responses of the person responding first if this person was the group member who had interacted with the confederate.

An interesting procedure in memory conformity research is the MORI technique (*Manipulation of Overlapping Rivalrous Images*; Mori, 2003, 2007), which consists of the simultaneous projection of two movie versions on the same screen. The two versions differ in terms of certain details; for example, in one version, the criminal checks the time on a wristwatch, while in the other, he does so on a wall clock. Thanks to the polarized glasses (which look like regular sunglasses) that are worn by the pair of participants, each of them sees a different movie version while being convinced that they are both actually watching the same movie clip. Thus, the participants sit beside each other, look at the same screen, and are not aware that the other person is watching a different version.

A disadvantage of the MORI technique is that it requires sophisticated technical equipment. To overcome this, Cadavid and Luna (2021) proposed a modification of this procedure called the MORI-v technique. Here, pairs of participants separately get acquainted with the different movie versions displayed on smartphone screens. Thus, polarized glasses are not necessary. Afterward, in order to introduce misinformation via an instant-messaging app, the experimenter sends multiple-choice questions concerning the movie, and the participants discuss them by chatting on the app. In the end, participants undergo a virtual individual recognition test on smartphones (Cadavid and Luna, 2021; Experiment 2). It should be stressed that regardless of which of the aforementioned procedures is used, the memory conformity effect is reliably replicated. Therefore, it is reasonable to ask about the mechanisms of this phenomenon, that is, the reasons why a given person succumbs to misinformation from a co-witness.

In the relatively scarce studies of the mechanisms of memory conformity, the classification proposed by Wright et al. (2009) is used most often. It is partly based on the distinction proposed by Deutsch and Gerard (1955) in the context of social influence and describes three processes which may cause the memory conformity effect. The first one is *normative influence*, which results from the need for social acceptance and avoiding the costs of disagreeing in social situations. It occurs because a person does not agree with information provided by an interlocutor but does not disclose their own opinion to avoid confrontation (Asch, 1956). It basically involves comparing the costs of disagreeing with the costs of making an error (Wright et al., 2010). This was confirmed in a study by Baron et al. (1996, Experiment 1), who manipulated the cost of an error: Two times as many participants yielded to misinformation when they were told that the experiment consisted of pilot data compared to participants who thought that the data would be used by police and courts and that the most accurate participants would be given a monetary prize. Also, Skagerberg and Wright (2008, 2009) showed that the perceived power of the partner influences memory conformity; this effect can be explained, for example, in terms of normative influence.

The second possible mechanism underlying memory conformity is *informational influence*. It refers to a situation in which a person succumbs to misinformation provided by an interlocutor as they are certain the interlocutor is right and is the source of more accurate information. While normative influence involves comparing the costs of disagreeing with the cost of errors, the informational impact is connected with comparing the relative likelihood of the other person being correct versus oneself being correct (Wright et al., 2009). In contrast to normative influence, which could be expected to be reduced to zero when the participants answer in private (as there are no costs of disagreeing in this situation), the informational impact may be present in this context as there is no social influence.

Informational influence can be expected to be related to the perceived accuracy of one’s own memory (as well as the partner’s), the credibility of the partner, and subjective confidence in one’s memory. Some research confirms these assumptions (Wright et al., 2000; Gabbert et al., 2007; French et al., 2011; Allan et al., 2012; Wright and Villalba, 2012; Williamson et al., 2013; Goodwin et al., 2017; Thorley and Kumar, 2017; Sousa and Jaeger, 2022; Kękuś et al., in press).

The third potential mechanism of memory conformity may be connected with memory distortion: The false information provided by other persons may influence the actual memories of the witness. Wright et al. (2009) differentiate between two possibilities: (1) just *believing* that the information provided by the partner is true or (2) the new information becomes part of episodic memory (as defined by Tulving, 1983). Believing that false information is true may also be related to source-monitoring errors; that is, the participant erroneously assumes that details mentioned by the partner were present in the original material (Oeberst and Seidemann, 2014).

The major aim of our research was to examine the effectiveness of a modified version of the MORI-v technique (Cadavid and Luna, 2021) in inducing the memory conformity effect. The original MORI technique is a useful tool for examining the memory conformity effect as—unlike other experimental procedures—it reduces the risk that participants will suspect manipulation, especially when they disagree on certain details during the discussion. In addition, the MORI technique is a good approximation of laboratory conditions to real-life situations where people witness a given crime, talk about it, and are then individually interviewed by enforcement agencies. However, this method requires special apparatus (projectors with polarizing filters, a translucent ground-glass screen, and special polarizing glasses), but not all researchers can afford such equipment. The MORI-v technique is an economical and readily available alternative to the original method. In addition, the MORI-v technique makes it possible to investigate the effects of virtual misinformation (Cadavid and Luna, 2021), which seems important given the increasing number of Internet users each year (Internet World Stats, 2023) and the increasing sharing of unverified information on social networks (Marsh and Rajaram, 2019).

Our basic idea was to make the MORI-v technique suitable for testing participants in full web-based settings instead of laboratories. In this method, participants are presented with original material on their own computers. Next, a pair of participants connected to the experimenter via an instant messenger with cameras and microphones on. The experimenter provides access to their computer screen so the participants can see a PowerPoint presentation with questions about the original material. As in the MORI and MORI-v techniques, the pair provides answers to 12 questions together, four of which refer to dissimilar details, thus leading to mutual misinformation. In contrast to the MORI-v, the participants talked to each other rather than writing their answers. In the end, participants undergo an individual recognition test on their own computers and are asked not to contact each other.

Apart from this, it is worth noting that both MORI-v and our modified online version of it may have some advantages over the original version. Namely, the original MORI procedure requires by design that the participants are told that the glasses will diminish their visual acuity. This could lessen their confidence in what they see. However, confidence in one’s memory is an important predictor of memory conformity (e.g., Wright et al., 2000; Wright and Villalba, 2012; Thorley and Kumar, 2017; Yue et al., 2021; Sousa and Jaeger, 2022; Kękuś et al., in press). Therefore, the original procedure may not be as “ecological” as in real life (and research using other methods), where witnesses’ confidence is not challenged by design.

Using the online MORI-v technique, we expected the usual memory conformity effect to arise; that is, the proportion of correct answers should be lower in cases in which the partner has mentioned details that are incongruent with what the participant actually saw. Apart from this, our second aim was to replicate the effect on memory conformity of disputing with the partner. In order to explain this, the distinctions between *discussed* and *non-discussed* details and between *disputed* and *non-disputed* ones should be presented.

The main factor in the MORI technique is that of *discussion* between the participants. This term refers simply to situations in which one participant gives answers inconsistent with what their partner saw. For example, one of the participants may have seen a black cap, while the second one sees a red one. If the first participant answers ‘black’, such a situation is classified as “discussed”. This means that the second participant has been misinformed [or misdirected, as Cadavid and Luna (2021) put it]. Now, such discussed (misinforming, misdirecting) details may be divided into disputed and non-disputed ones (see Cadavid and Luna, 2021, Table 1; Kękuś et al., 2023, Figure 1). A given detail is classified as disputed if the participants in a pair disagree with each other during the discussion about it and give different answers (but consistent with their own original information). On the other hand, if, when discussing a dissimilar detail, one participant gives an answer that is consistent with their own original material and the other participant does not dispute the answer, the detail is classified as non-disputed. Thus, disputed details are equated with *mutual* misinformation, which means a pair of participants provide misleading information to each other, while non-disputed details are equated with *unilateral* misinformation, which means one participant misinforms the other (Ito et al., 2019). In existing research using the classic MORI technique, it has been found that when participants dispute a given detail, that is, they dispute what their partner said, they are less likely to be misled (French et al., 2008; Garry et al., 2008; Ito et al., 2019). The same was expected in the present research, not only because it has already been found in existing research but also because it makes logical sense. If the participant does not question their partner’s answer, this might be an indication that they are convinced that the ‘information’ from the partner is the correct answer to a given question. This may be the case even if their own memory is different. This would mean that informational influence is at play.

Lower correctness in the case of non-disputed compared with disputed details may also arise because of the third of the mechanisms mentioned by Wright et al. (2009), namely memory distortions. Failing to question what the partner said may be an indication that a participant indeed “remembers” the misinformation provided by the partner as if it were their own.

As for the normative impact, it was not considered important in the present study as the final recognition test was performed by the participants individually; therefore, there was no risk of confrontation in this situation.

The third aim of our research was to analyze the relationship between succumbing to misinformation and three individual characteristics: susceptibility to social influence, need for closure, and self-esteem. Susceptibility to social influence was measured by means of a self-description questionnaire called *Measure of Susceptibility to Social Influence* (MSSI; Bobier, 2002), described in detail below. In this tool, three dimensions of susceptibility to social influence were assumed: *Principled Autonomy*, reflecting independence of judgement and beliefs; *Social Adaptability*, referring to compliance with others, that is, allowing oneself to be influenced in order to avoid confrontation; and *Social Friction*, which can be defined as “anticonformity”. It was hypothesized that all these three dimensions would be related to memory conformity: Non-conformist people who are independent in their judgments and opinions and are ready to engage in confrontation should rely on their own recollections rather than on information from their partner. In the existing literature, at least one result has been shown to be promising when searching for correlations between memory conformity and other types of suggestibility, namely the positive relationship that has been found between memory conformity and interrogative suggestibility (Thorley, 2013). Interrogative suggestibility (Gudjonsson, 1997) refers to the tendency to answer in accordance with suggestions contained in misleading questions (Yield) and the tendency to change one’s answers under the influence of negative feedback (Shift). Memory conformity has been shown to be correlated with Yield but not Shift (Thorley, 2013). In other research, compliance, as measured by the Gudjonsson Compliance Scale (GCS, Gudjonsson, 1997), was positively related to memory conformity (Merckelbach et al., 2007).

Need for closure (NFC) is a construct described by Webster and Kruglanski (1994). It refers to an individual’s desire for firm answers and their aversion to ambiguity. People with high NFC have a strong need to reduce the feeling of discomfort experienced in the face of cognitive uncertainty through the quick formulation and validation of a hypothesis. NFC includes five facets: Preference for order, Predictability of future, Decisiveness, Discomfort with ambiguity, and Closed-mindedness. We expected all these to be related to memory conformity as they imply a kind of difficulty with ambiguities, and ambiguities are a natural element of the memory conformity paradigm—when what the partner said is inconsistent with what the participant saw. People with high NFC may be tempted to resolve ambiguities by simply assuming that they are wrong and that their partner is correct. Therefore, our hypothesis was that it would correlate positively with memory conformity.

Finally, we expected self-esteem (SE) to be negatively related to memory conformity because low self-confidence is related to it (e.g., Wright et al., 2000; Wright and Villalba, 2012; Thorley and Kumar, 2017; Yue et al., 2021; Sousa and Jaeger, 2022; Kękuś et al., in press), and self-confidence is, in turn, related to self-esteem (e.g., Campbell, 1990; Coudevylle et al., 2011).

To sum up, the aims of the present study were as follows: (1) to replicate the memory conformity effect by means of the online version of the MORI-v technique, (2) to analyze the difference in correctness between disputed and non-disputed items, and (3) to analyze the correlations between memory conformity and susceptibility to social influence, need for closure, and self-esteem.

## 2 Method

### 2.1 Power analysis

The power analysis was performed using the G*Power software (Faul et al., 2009), assuming a desired power of 80%. A repeated-measures ANOVA was used for the analyses (for the main effect of memory conformity, as well as for the differences between disputed and non-disputed items). Also, power was calculated for Pearson’s *r* correlations for the hypotheses concerning the relationship between memory conformity and individual traits.

As for the repeated-measures ANOVA, for the three effect sizes typically considered as small, medium, and large, that is, *f* = 0.1, 0.25, and 0.40, respectively (Cohen, 1988), the required sample sizes were 787, 128, and 52. In fact, in existing research across 10 countries, *f* was much higher: about 0.95 (translated from Hedges’ *g* = 1.92, reported by Ito et al., 2019). As for correlations, the required sample sizes for small, medium, and large effects (*r* = 0.10, 0.30, and 0.50) are 783, 85, and 29, respectively.

Given the existing resources, a sample size of about 60 participants was assumed. This assured satisfactory power in the case of medium–to-large effect sizes but not for small ones.

### 2.2 Participants

A total of 72 participants (36 pairs, 44 women and 28 men) recruited via social media took part in the research. The mean age of participants was 25.0 (SD = 3.87). The youngest person studied was 18 years old, and the oldest was 54 years old. The participants signed a written informed consent. The Research Ethics Committee at the Faculty of Philosophy of the Jagiellonian University had no objections to the research.

### 2.3 Materials and design

#### 2.3.1 Modified MORI-v technique

In general, the MORI and MORI-v techniques consist of the following main elements: (1) original material, that is, two versions of the film; (2) the collaborative recognition test, that is, a discussion about the original material, which leads to mutual misinformation), and (3) an individual recognition test that checks the effect of the discussion on the memory account of the original material. These elements are fully derived from the MORI technique and are described below.

##### 2.3.1.1 Original material

The original material (used in previous studies in terms of the MORI technique, for example, Garry et al., 2008; Ito et al., 2019) was a silent movie lasting 6 min and 34 s. The recording depicts an electrician (“Eric”) who steals some objects while repairing various household appliances. The movie was created in two versions by Takarangi et al. (2006). The versions are identical except for eight critical details; for example, in one version, Eric puts on a red cap, but in the other version, it is black.

##### 2.3.1.2 The collaborative recognition test

This consisted of a series of questions, thanks to which the participants discussed half of the discrepant details included in the original material, thus leading to mutual misinformation. The test comprised 12 questions, four of which refer to critical details (e.g., “what color was Eric’s cap?”), with the other eight being control questions. Each question had five possible responses and was displayed in a PowerPoint presentation for 60 s while participants discussed the answer. If the participants were unable to reach an agreement, both responses were documented. The discussion was audio recorded.

##### 2.3.1.3 Individual recognition test

The test included 20 questions, with eight of them referring to all critical details included in the original material. The test was used to compare the proportion of correct answers to the questions concerning the discussed details (related to misinformation) against the proportion of correct answers to the questions concerning the non-discussed details.

#### 2.3.2 Individual differences questionnaires

- *The Measure of Susceptibility to Social Influence* (MSSI; Bobier, 2002; Polish adaptation: Polczyk, 2007) consists of 34 statements that evaluate the tendency to succumb to social influence. The tool consists of three subscales: Autonomy, Social Adaptability, and Social Resistance. The answer to each question is ranked on a scale of 1–5.
- *The Need for Closure Scale* (NFCS; Webster and Kruglanski, 1994; Polish adaptation: Kossowska et al., 2012). The shortened version of this tool consists of 15 statements to which the subjects respond on a 6-point scale. The tool includes the following five subscales: Preference for order, Predictability of future, Decisiveness, Discomfort with ambiguity, and Closed-mindedness.
- *Self-Liking/Self-Competence Scale-Revised* (SLCS-R; Tafarodi and Swann, 2001; Polish adaptation: Szpitalak and Polczyk, 2015) consists of 16 statements measuring two dimensions of self-esteem: self-competence and self-liking. The answer to each question is ranked on a scale of 1–5.

### 2.4 Procedure

Participants were examined in pairs during a single experimental session conducted via the Internet. They were informed that the research concerns the social sharing of information. In addition, they were instructed to use a computer, laptop or tablet rather than a smartphone during the study.

First, participants received a link to the movie, which was the original material. For each person in each pair, the movie was identical except for eight critical details. After watching the movie, participants were asked to complete The Need for Closure Scale and the Self-Liking/Self-Competence Scale, which provided an additional interval between the presentation of the original material and the introduction of the misinformation. Next, the subjects were requested to launch the Skype application. This part began with an instruction given by the experimenter, which was taken from research by Garry et al. (2008) and modified as needed:

“Thank you for participating in the first part of the experiment. I will audio-record our conversation with your permission.

In a moment, I will show you a series of questions about the movie you watched. You will see each question for 60 s. Ten seconds before that time expires, I will ask you for your answer.

You can also talk to each other during this time. If you have no idea what the answer is, talk to each other about the part of the movie the question is about or what was also happening in the movie at that time. This will help you remind each other of the right answer.”

Afterward, a series of 12 questions about the original material was shown to the participants using the screen-sharing function. During the discussion of the four critical details, participants inadvertently introduced misinformation. If the pair disagreed with each other on the answer to a given question, the experimenter noted both answers. During the discussion, the experimenter and the participants could see each other via webcams.

In order to counterbalance the discussed details, 50% of the pairs discussed different critical details than the other 50% of the pairs. For example, half the pairs talked about Eric’s cap color without discussing his company logo (version A of the discussion), while the other pairs discussed the logo but not the cap color (version B of the discussion). After the discussion, participants were asked to fill out The Measure of Susceptibility to Social Influence. Eventually, participants underwent the individual recognition test and were asked not to contact each other. In addition, the experimenter temporarily deactivated the link to the movie. After the experiment, participants were debriefed.

## 3 Results

The memory conformity effect was manipulated as a within-subject variable. The proportion of correct answers in the individual recognition test to questions about non-discussed details was compared with that of answers to questions about discussed (misinformed) details. In the case of non-discussed items, it was calculated as the proportion of correct answers to all non-discussed questions. In the case of discussed items, the score was calculated as the proportion of correct answers to questions about details for which a given participant received misinformation from the partner. Participants who were not exposed to any misinformation were excluded from the analysis, which left a sample of 66 subjects.

The results were clear-cut: the proportion of correct answers to questions about non-discussed details was much higher (*M* = 0.74, SD = 0.23) than for the questions about discussed and misinformed details (*M* = 0.38, SD = 0.36; *F*(1, 65) = 54.06, *p* < 0.001, η^2^ = 0.45, Hedges’ *g* = 1.17, 95% CI [0.68, 1.69]). Thus, the effectiveness of the modified version of the MORI-v technique (Mori, 2003; Cadavid and Luna, 2021) in inducing the memory conformity effect was confirmed. Moreover, the size of the effect was large.

In the second analysis, the proportion of correct answers in the individual recognition test for details that were disputed and non-disputed during the discussion was computed. This analysis was performed for participants who disputed an item at least once (*n* = 36). The proportion of correct answers to questions about disputed details was much higher (*M* = 0.84, SD = 0.33) than for non-disputed ones (*M* = 0.17, SD = 0.38; *F*(1, 35) = 51.72, *p* < 0.001, η^2^ = 0.60, Hedges’ *g* = 1.84, 95% CI [1.28, 2.47]). Thus, it was demonstrated that if one participant introduced misinformation, and the other agreed with it during the discussion about a particular item, the latter’s answer about that item during the individual recognition test was most often incorrect. However, if one participant introduced misinformation and the other protested during the discussion about an item, the latter’s answer about that item during the individual recognition test was most often correct.

In the last set of analyses, individual variables (susceptibility to social influence, need for closure, and self-esteem) were correlated with resistance to the memory conformity effect. The latter was calculated as the difference between the proportions of correct answers to questions about non-discussed details minus the proportion of correct answers to discussed ones. This means that the higher the mean proportion, the better the accuracy when non-discussed and discussed items are compared; in other words, the better the result, the higher the resistance to misinformation. The results are presented in Table 1.

**Table 1:** Correlations between individual variables and resistance to memory conformity.

Questionnaire	Subscale	Resistance to memory conformity
Measure of susceptibility to social influence	Principled autonomy	0.25*
	Social adaptability	−0.35**
	Social friction	0.05
Need for closure	Preference for order	−0.16
	Predictability of future	−0.01
	Decisiveness	0.01
	Discomfort with ambiguity	−0.27*
	Closed-mindedness	−0.05
Self-liking—self-confidence	Self-liking	−0.17
	Self-confidence	−0.11

*: *p* < 0.05. **: *p* < 0.01.

The results indicate that the higher the Principled autonomy and the lower the Social adaptability, the higher the resistance to memory conformity. In other words, participants who were autonomous were resistant to misinformation, while those who preferred to adapt to social rules were less resistant to misinformation. In addition, higher discomfort with ambiguity was connected with lower resistance to misinformation. The remaining correlations were not statistically significant.

## 4 Discussion

The major aim of our research was to examine the effectiveness of a modified version of the MORI-v technique (Mori, 2003; Cadavid and Luna, 2021) in inducing the memory conformity effect. Obviously, the details of our technique differ from the MORI and MORI-v techniques, but the main idea was preserved Two participants thought that they were watching the same movie while, in fact, it differed in some details. As a result of this, while discussing the movie, the participants mutually misinform each other.

Our assumption was confirmed: The proportion of correct answers concerning discussed and misinformed details was much lower than for non-discussed ones. The size of the effect (Hedges’ *g* = 1.17) was large and comparable to other studies. For example, Ito et al. (2019) reported effect sizes obtained using the classic MORI technique from 11 countries. They ranged from 1.01 (Colombia) to 2.97 (Japan). As for Poland, the effect size reported by Ito et al. (2019) was 1.92, with 95% CIs: 1.28, 2.56. It may conclude that the online version of the MORI-v technique generates memory conformity of comparable size to the classic version of this technique.

Thus, the online version of the MORI-v technique may be a useful and handy method for studying memory conformity. Online research may be, for example, the only option during a lockdown caused by a pandemic. In addition, web-based procedures make it possible to obtain a much larger and more diverse sample than most offline studies (Reips and Musch, 2002; Birnbaum, 2004) because Internet studies provide an opportunity to reach participants living in small towns and villages far from university laboratories, which are mostly located in large cities. Moreover, our method can be used to examine individuals who may have difficulty getting to the laboratory, for example, computer-literate disabled persons or seniors. What is more, as virtual contact occurs under relatively ‘safe’ conditions, it gives the potential to reveal behaviors that would likely be inhibited in face-to-face contact. Online research reduces symptoms of shyness and minimizes fear of evaluation by the experimenter, which is a major problem in laboratory research (Rosenberg, 1965; Dzwonkowska, 2003; Grzyb, 2017).

The hypothesis referred to the distinction between disputed versus non-disputed details, that is, the manner in which participants discuss the original material. It was assumed that the proportion of correct answers would be higher for questions about disputed details than for non-disputed ones. This hypothesis was confirmed. This result is also congruent with existing research using the classic MORI technique in which the proportion of correct answers to questions concerning disputed details was higher than that for non-disputed details (French et al., 2008; Garry et al., 2008; Ito et al., 2019). Thus, this effect was also replicated in the present research using the modified MORI-v technique.

The fact that disputing an item, that is, raising doubts about given misinformation, results in a higher proportion of correct answers might be caused by different factors. It is possible that a given participant had their own correct memories about a given item, and this fosters a correct answer. However, failing to dispute (i.e., to question) certain misinformation does not mean that the participant did not remember anything about a given item (although this is possible). It could be that relevant and correct memories were present, but the participant failed to disclose that they remembered something different than their partner as they were certain the interlocutor was right. In research by Kękuś et al. (in press), up to 58.3% of participants who had different memories from their partners yielded to misinformation and reported afterward that the reason for this was lack of confidence in their own recollections. In another 41.7% of cases, the participants declared that they preferred to trust information provided by their partners, thinking that their partners “knew better.” In sum, informational influence might also have occurred in the present research.

Our next hypothesis concerned the expected relationships between memory conformity and susceptibility to social influence, the need for closure, and self-esteem. These traits were measured by means of self-descriptive questionnaires. As for susceptibility to social influence, two of its dimensions proved to be related to succumbing to misinformation: Principled autonomy was related to higher resistance to misinformation, while Social adaptability was related to lower resistance. Social friction was not significantly statistically related to yielding to misinformation received from the other person. The hypothesis that relationships would be found between general susceptibility to social influence and yielding to misinformation was confirmed, at least in two out of the three facets of susceptibility to social influence. This could mean that the memory conformity effect might be just a kind of susceptibility to social influence. This would be logical as memory conformity is a kind of social influence, after all. However, this result should be taken with caution as the correlations between memory conformity and the same three dimensions of social influenceability have proved nonsignificant in other similar analyses (Kękuś et al., in press).

As for the need for closure, the results were not very compelling: Out of its five dimensions, only higher discomfort with ambiguity was related to lower resistance to misinformation. As for self-esteem, no significant correlations were obtained. Thus, the hypothesis relating to these traits should be treated as not confirmed.

## 5 Limitations and future directions

The present research was conducted online. Such research is convenient for researchers and has advantages, for example, access to a research sample with a wide range of psychodemographic parameters (Mason and Suri, 2012). However, the modified MORI-v technique is not without disadvantages, including difficulties with controlling various variables, including the main ones. It is possible that the motivation of some participants was not high; this could result in, for example, not paying sufficient attention to the original material, which in turn might artificially augment the memory conformity effect as a result of a lack of memories of the original details.

In future research on memory conformity, it might be useful to apply open-ended questions in the individual test. Such a form of questions might be more ecologically valid as questions in the form of closed alternatives should be avoided in real interrogations. Questions of this kind have already proved promising in research on memory conformity (Kękuś et al., in press).

## Data availability statement

The original contributions presented in the study are included in the article/Supplementary material, further inquiries can be directed to the corresponding author.

## Ethics statement

The studies involving humans were approved by Research Ethics Committee at the Faculty of Philosophy of the Jagiellonian University, Kraków, Poland. The studies were conducted in accordance with the local legislation and institutional requirements. The participants provided their written informed consent to participate in this study.

## Author contributions

All authors listed have made a substantial, direct, and intellectual contribution to the work and approved it for publication.
